# The Relationship Between Menopause and Dysphagia: A Scoping Review

**DOI:** 10.1089/whr.2022.0078

**Published:** 2022-12-15

**Authors:** Charles Lenell, Rodolfo Peña-Chávez, Ryan J. Burdick, Nicole Rogus-Pulia

**Affiliations:** ^1^ Department of Communication Sciences and Disorders, University of Northern Colorado, Greeley, Colorado, USA.; ^2^ Department of Communication Sciences and Disorders, University of Wisconsin-Madison, Madison, Wisconsin, USA.; ^3^ Department of Medicine-Geriatrics and Gerontology, University of Wisconsin-Madison, Madison, Wisconsin, USA.; ^4^ Departamento de Ciencias de la Rehabilitación en Salud, Facultad de Ciencias de la Salud y de los Alimentos, Universidad del Bio-Bio, Concepción, Chile.; ^5^ Geriatric Research Education and Clinical Center (GRECC) Veterans Administration Hospital, William S. Middleton Memorial Hospital, Madison, Wisconsin, USA.; ^6^ Otolaryngology—Head and Neck Surgery, Department of Surgery, University of Wisconsin, Madison, Wisconsin, USA.

**Keywords:** swallowing, dysphagia, menopause, scoping review, hormones

## Abstract

**Purpose:**

Menopause marks the end of fertility and rapid decline of ovarian hormones in the female body, which corresponds to a myriad of changes to bodily systems, including the upper aerodigestive tract. Despite substantial evidence that menopause negatively impacts oral health, bones, and skeletal muscles, little research has examined these effects as they relate to swallowing. The purpose of this scoping review was to compile and summarize the existing literature investigating the relationship between menopause and swallowing-related structures and physiology.

**Methods:**

Search terms were selected for three databases (PubMed, Scopus, and CINAHL) to gather relevant literature evaluating the relationship between menopause and swallowing-related anatomy as well as swallowing functions in both human and animal models. Relevant articles were reviewed, collated, and summarized to synthesize findings, identify gaps in the literature, and provide suggestions for future directions.

**Results:**

This scoping review yielded 204 studies with the majority of these studies relating to one or more of the following categories: oral health, saliva, mandibular structures, and taste. Common oral symptoms reported in the literature included xerostomia, hyposalivation, tooth decay, inflammation of oral mucosa, and oral pain. Although literature supports that menopause adversely affects oral health, saliva, mandibular structures, and alters taste, a dearth of information was evident regarding how these hormone-dependent changes can adversely affect swallowing.

**Conclusions:**

The relationship between menopause and swallowing has been overlooked by field of speech–language pathology. By identifying the major gaps in the literature, these results will inform future investigations evaluating relationships among ovarian hormones and swallowing.

## Introduction

Menopause marks the end of the female reproductive cycle, results in the dramatic loss of ovarian hormones, and occurs at the median age of 51.5 years in the United States.^1^ This drastic change in the endocrine system results in myriads of changes in the female bodily systems, including the upper aerodigestive system. Although menopause has been reported to negatively affect bone density,^2^ oral mucosa and health,^3^ and skeletal muscles,^4,5^ the relationship between ovarian hormones and the swallow mechanism is poorly understood. Therefore, the purpose of this scoping review article is to summarize pertinent literature that highlights the relationship between menopause and swallowing-related structures/functions.

Although menopause occurs during middle age, menopausal dysphagia symptoms likely differ from presbyphagia, age-related changes to the swallow mechanism, or sarcopenic dysphagia, an age-related loss of whole-body muscle mass leading to dysphagia.^6,7^ Both presbyphagia and sarcopenic dysphagia occur in older adults (65+ years old), whereas perimenopause occurs approximately two decades prior. Additionally, menopause-related changes to the oral cavity and oral health^3,8,9^ that could negatively affect swallowing are likely due to the rapid decline of ovarian hormones, rather than the aging musculoskeletal system like with presbyphagia and sarcopenic dysphagia. The following paragraphs will summarize how menopause adversely affects structures and functions related to swallow physiology, which serve as a rationale for conducting the scoping review.

The loss of estrogen (estradiol) during and after menopause adversely affects bone mass and microarchitecture leading to a greater prevalence of osteoporosis in aged women compared with aged men.^10,11^ In fact, estrogen is considered the key regulator of bone metabolism (remodeling and reabsorption) in both men and women.^12^ Thus, menopause could potentially negatively affect bones that are important in the swallowing process (*i.e.*, mandible, hard palate, hyoid).

In addition to structural effects, the loss of ovarian hormones during menopause has also been associated with adverse changes to oral health, including saliva production and composition, periodontal health, and oral discomfort symptoms.^8^ These oral changes and symptoms may impact swallowing function. For example, hyposalivation (decreased quantity of saliva) leads to xerostomia (perception of dry mouth), a common oral symptom of postmenopausal women.^8,13^ Xerostomia is related to greater perceived swallowing impairment.^14,15^ Hyposalivation and salivary compositional changes result in poor bolus lubrication resulting in swallowing inefficiency.^16,17^ Poor oral health leads to inadequate dentition, which affects mastication and increases the risk for asphyxiation.^18–20^ However, complex relationships among ovarian hormones, oral health, and swallowing perception have not been fully elucidated.

Menopause also adversely affects muscle structures and functions.^4,5,21,22^ The role of estrogen on skeletal muscle function is important for muscle repair and response to exercise.^23^ Nevertheless, during perimenopausal years, women experience an accelerated loss of muscle mass and performance, primarily attributed to the preferential atrophy of fast-twitch muscle fibers.^22^ Animal studies have also demonstrated adverse effects of menopause on the size and performance of both fast-and slow-twitch muscles.^22,24,25^ Because swallowing involves the complex orchestration of cranial nerves and skeletal muscles,^26^ menopausal effects on swallowing-related musculature could lead to swallowing impairments. Nevertheless, few studies have evaluated the effects of menopause on swallowing-related muscles.

In summary, menopause appears to affect several structures and functions important for safe and efficient swallowing; however, the strength and nature of these relationships are currently unclear. Therefore, the goal of this scoping review was to compile and summarize evidence regarding the relationship between menopause and swallowing in the following areas: swallowing-related structures, oral health and symptoms, and swallowing functions. Additionally, for each domain, gaps and future directions were outlined.

## Methods

A protocol was developed before initiating the literature search to minimize bias in the eventual findings of the review. The protocol established and documented the following sections: (1) identify the research question; (2) identify relevant studies and develop a decision plan for sources, terms, time frame, and languages; (3) study selection using predetermined inclusionary and exclusionary criteria; (4) chart the data; and (5) collate, summarize, and report the results.^27,28^ For step 1, the overall research question was “What is the relationship between menopause and swallowing-related structures/functions?”

For step 2, the authors consulted a university librarian to identify the relevant studies using appropriate databases and search terms. The terms included in the scoping review are reported in Table 1 for the utilized databases: PubMed, Scopus, and CINAHL. Menopause-related terms were selected to focus the review on changes to swallowing specific to menopause rather than age-related swallowing changes; therefore, no age-related terms were included in this scoping review. The languages of the studies were limited to English. The time frame was 1980–2020 based on the timing of integration of dysphagia management into the speech–language pathologist's scope of practice.^29^

**Table 1.** A Categorized List of Swallow-Related Terms Included in the Scoping Review

Inclusion categories	Menopause or swallow-related terms	Search terms
Menopause	Menopause Ovariectomy Oophorectomy	Menopause Menopausal Perimenopause Ovariectom^*^ Oophorectomy “Menopause, Premature” “Premenopause” “Postmenopause” “Hysterectomy” “Hysterectomy, Vaginal”[Mesh] “Ovariectomy”[Mesh] “Salpingo-oophorectomy”[Mesh] Hysterectomy[tw] Menopaus^*^[tw] “Low estrogen”
Swallow structures	Bones: Cervical vertebrae Hyoid Mandible Palate Muscles: Cricopharyngeus Infrahyoid muscles Mastication Pharynx Suprahyoid muscles Tongue Upper esophageal sphincter	“Cervical vertebrae”[tw] Hyoid[tw] Mandible[tw] Palate[tw]) Cricopharyngeus[tw] “Infrahyoid muscles” [tw] Mastication[tw] Pharynx[tw] “Suprahyoid muscles”[tw] Tongue[tw] “Upper esophageal sphincter”[tw]
Oral health and symptoms	Oral health: Mouth Mouth mucosa Oral mucosa Saliva Saliva composition Salivary flow Salivary glands Oral symptoms: Burning mouth syndrome Globus Hyposalivation Taste Xerostomia	Mouth[tw] “Mouth mucosa”[tw] “Oral mucosa”[tw] Saliva[tw] “Saliva composition”[tw] “Salivary flow”[tw] “Salivary glands”[tw] “Burning mouth syndrome”[tw] Globus[tw] Hyposalivation[tw] Taste[tw] Xerostomia[tw]
Swallow functions and dysphagia	Swallowing: Deglutition Laryngeal closure Laryngeal elevation Swallow Swallowing Dysphagia: Aspiration Aspiration pneumonia Deglutition disorder Dysphagia Laryngeal penetration Odynophagia	Deglutition[tw] “Laryngeal closure”[tw] “Laryngeal elevation”[tw] Swallow[tw] Swallowing[tw] Aspiration[tiab] “Aspiration pneumonia”[tw] “Deglutition disorder”[tw] Dysphagia[tw] “Laryngeal penetration”[tw] Odynophagia[tw]

For step 3–5, a decision plan was implemented to screen and categorize the studies. Initially studies were imported for screening; using Covidence software,^30^ duplicates were automatically removed; and the titles of studies were screened by three reviewers (speech–language pathologists). If the title did not relate to the overall research question, the study was excluded. Of note, because alveolar and mandibular bone changes are relevant to both the areas of dentistry and osteoporosis, we excluded articles focusing solely on the parameters of osteoporosis, dentition, and periodontal tissues with no explicit link to aspects of mastication or swallowing. Additionally, studies that evaluated the effects of menopause on salivary biomarkers unrelated to swallowing were also excluded. No studies were excluded based on experimental design. After titles were screened, the abstracts were screened independently by two reviewers.

If the abstract was relevant to the overall research question, the study was categorized into one of the following categories: alveolar bone, disorders/syndromes, esophagus, saliva, oral mucosa, mandible/temporomandibular joint (TMJ), nutrition/hydration, taste, tongue muscles, pharyngeal muscles, or saliva production. Full texts were reviewed if the appropriate determination of the relevance and category could not be made based on the abstract. If a discrepancy existed between the two reviewers, the lead author made the final decision for including and categorizing the study.

## Results

**FIG. 1.**
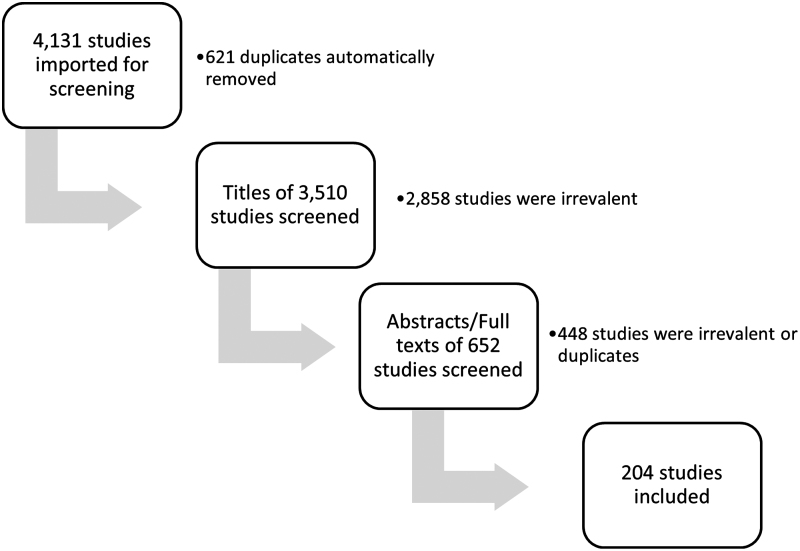
Studies screened for scoping review.

Of the total 4131 studies imported for screening, 204 studies were relevant to this scoping review (Fig. 1). A breakdown of all the major categories for each of the included articles is summarized in Table 2. Included articles were primarily related to oral health: oral mucosa; salivary composition, flow, or gland function; and xerostomia/hyposalivation. Of note, studies evaluating the role of ovarian hormones specifically on dental health (*e.g.*, dental caries) or periodontal tissues (*e.g.*, gingivitis) were not included in this scoping review; however, the relationship between dental and periodontal health and menopause has been established in the field of dentistry and comprised many excluded articles.

**Table 2.** Characteristics of the Studies Included in the Review (*n* = 204)

	*N*	%
Study design (*n* = 204)		
Animal models	94	46.1
Human studies	88	43.1
Reviews	18	8.8
Cell models	2	1.0
Retrospective studies	2	1.0
Animal model species (*n* = 94)		
Rat	74	78.7
Mouse	14	14.9
Rabbit	3	3.2
Monkey	2	2.1
Dog	1	1.1
Human study designs (*n* = 88)		
Case–control study	27	30.7
Cross-sectional study	26	29.5
Clinical trial	16	18.2
Case study	13	14.8
Cohort	4	4.5
Randomized control trials	2	2.3
Article category for rat studies (*n* = 74)		
Saliva composition, flow, or structures	25	33.8
Mandible/temporomandibular joint	22	29.7
Taste	16	21.6
Oral mucosa	5	6.8
Tongue muscle	3	4.1
Alveolar bone	2	2.7
Nutrition/hydration	1	1.4
Article category for human studies (*n* = 88)		
Saliva composition, flow, or structures	28	31.8
Oral mucosa	19	21.6
Disorders/syndromes	15	17.0
Xerostomia	11	12.5
Mandible/temporomandibular joint	5	5.7
Taste	5	5.7
Esophagus	2	2.3
Tongue muscle	1	1.1
Pharynx	1	1.1
Globus	1	1.1

Overwhelmingly, the studies highlighted the relationship between menopause (loss of ovarian hormones) and negative changes to oral health. Common menopause-related oral health signs/symptoms included xerostomia, hyposalivation, inflammation of oral mucosa, and oral pain.^3,8,9,31^ Additionally, both salivary flow rate and saliva composition were found to be affected by menopausal status. The primary reported changes to saliva postmenopause included lower salivary pH levels and decreased salivary flow rates.^32–37^

Menopause was found to negatively affect both the alveolar bone and the mandible or TMJ. Although many studies evaluated the effects of menopause on these alveolar and mandibular bones, a majority were excluded due to the focus on dental, osteopenia, and/or osteoporosis outcome measures. Many included studies investigated how the loss of ovarian hormones and reduced masticatory activity negatively impacted the mandible and TMJ.^38–42^

Twenty-four studies were relevant for evaluating the relationship between ovarian hormones and taste. A major finding from these studies suggested that menopause influenced sucrose palatability and resulted in a reduced perception of sucrose taste consequently altering eating habits to prefer sweater food.^43–46^ Studies also demonstrated sex differences between tastes and found females to prefer sweeter foods, which is likely mediated by gonadal hormones.^46,47^

Finally, some studies involved describing ovarian hormone-related syndrome/disorders and swallowing structures. For example, several case studies were present in the literature regarding female cancers (*i.e.*, breast, cervical, uterine, etc.) that metastasized to swallowing structures.^48–52^ Also, certain female-predominant syndromes, such as Sjogren's syndrome and Burning Mouth Syndrome are known to affect salivary gland function and oral health.^14,53,56^

## Discussion

Although menopause is known to negatively affect oral health, oral mucosa, bone density, and skeletal muscle structures/functions, this scoping review highlights the dearth of information regarding how menopause may negatively affect swallowing structures and consequently swallow functions. To date the literature that evaluates the relationship between swallowing structures and menopause is primarily in the field of dentistry. As a result, although the effects of postmenopausal changes to salivary gland functions (*i.e.*, hyposalivation and xerostomia) have been well documented to impact dental and periodontal tissue health, the impact on swallow functions and perception remains unknown.

This review yielded extensive literature connecting menopause to disturbances in oral health, including xerostomia, reduced salivary flow, and burning mouth syndrome. Additionally, although not discussed in this review, menopause is known to adversely affect both dental and periodontal health as well.^8,33,57,58^ However, these disturbances in oral health were not examined as they are relevant to dysphagia and its consequences. In fact, no identified research examined the relationship between menopausal status of individuals with dysphagia and aspiration pneumonia, despite the well-established relationship between poor oral health and the development of aspiration pneumonia, especially among older adults.^59,60^

Because menopause negatively affects oral hygiene, future research should investigate the relationship between menopausal status and aspiration pneumonia risk in individuals with dysphagia. Considering the results of this review, future studies should evaluate if menopause may exacerbate oral dysbiosis such that postmenopausal individuals with dysphagia are more likely to experience aspiration pneumonia than premenopausal individuals with dysphagia.

Given that estrogen is the key hormone responsible for bone mass maintenance,^12,61^ menopause is known to adversely affect the structure of the mandible, alveolar bone, and TMJ.^62–67^ Therefore, literature investigating the effects of menopause on mastication was expected. Nevertheless, studies that investigated the relationship between menopause and these structures focused on how the loss of ovarian hormones and reduced masticatory activity negatively impacted mandibular structures, rather than how changes in these bony structures result in disturbances in mastication.^38–42,62–66^ Therefore, a clear gap in the literature exists regarding how menopause may negatively impact mastication.

Several syndromes with female predominance such as Sjogren's syndrome and Burning Mouth Syndrome were found to negatively impact oral mucosa and oral health and be influenced by ovarian hormones.^53,68^ Additionally, some female-specific cancers (*i.e.*, cervical, uterine, endometrial) were found to metastasize to the head and neck structures and in some cases result in dysphagia.^48,50,51^ In fact, a meta-analysis from a review article found that hormone replacement therapy reduced the risk of head and neck cancers in women.^69^ Future research should evaluate how hormonal status may affect swallowing perception, swallowing outcomes, and lung status for women with female-predominant or female-specific syndromes or disorders that affect swallowing structures.

This review additionally revealed a small group of literature examining the effect of menopause on the tongue in both animal models^70–75^ and humans.^76^ Current evidence shows that tongue activity is sensitive to ovariectomy suggesting that female hormones may have a substantial impact on lingual function. Using estradiol as replacement treatment in ovariectomized monkeys and rats, the activity of the tongue was modified when the level of the hormone was restored. For example, dyskinesis of the tongue was reduced,^73^ and also increased^74^ in ovariectomized monkeys. In ovariectomized rats, estradiol increased the fatigue resistance of genioglossus muscles.^71^ Same findings were found using phytoestrogens (genistein) demonstrating that estrogen hormones could improve the endurance of the genioglossus muscle in ovariectomized rats.^72^ In the case of reduced level of estrogen, Liu et al,^75^ found in ovariectomized rats that the contractile and electromyography (EMG) activity of the genioglossus activity was significantly decreased.

This trend was also found in postmenopausal women who showed lowest genioglossus activity. However, after 2 weeks of combined estrogen and progesterone replacement therapy in standard doses, a significant increase in EMG activity of the genioglossus was seen.^76^ Therefore, a clear effect of female hormones on tongue muscle activity has been reported as a consequence of menopause. Future research should evaluate the effects of menopause-related lingual changes on swallowing and the potential to reverse these adverse changes with therapeutic exercise.

The results of this review do not provide sufficient evidence to disentangle aging from menopause in human studies. The focus of this article was the impact of menopause on swallow structures and functions, which coincides naturally with aging. However, the results of this scoping review can be attributed to hormones rather than aging for two primary reasons. First, “aged” is typically evaluated at 65+ years of age, whereas menopause occurs ∼15 years earlier, so while nearly all aged women (65+ years old) are postmenopausal, postmenopausal women are not necessarily “aged.” Many human studies that evaluate menopause do not include aged individuals. Second, animal studies that ovariectomized young female animals to surgically induce menopause showed similar findings further corroborating these findings are related to hormones and not overall age. Nevertheless, the interaction of age and hormone status is likely important and was not directly investigated in this study.

In summary, from this review, menopause negatively affected oral health, saliva, the mandible/TMJ, and taste. Additionally, several female-predominant disorders and syndromes negatively affected oral health and/or swallowing structures. These findings clearly highlight relationships among ovarian hormones and swallowing structures likely impacting swallowing functions and suggest that menopause may affect swallowing structures/functions placing postmenopausal women at greater risk for dysphagia than premenopausal women. Future studies are needed to elucidate the effects of ovarian hormone shifts related to menopause in women on swallowing-related functions.

## Conclusion

Although menopause is known to adversely affect structures and functions related to swallowing such as bone, muscle, and mucosa of the upper aerodigestive tract, saliva, and taste, the relationship between menopause and swallowing functions remains poorly understood. By identifying the major gaps in the literature, these results will help inform future investigations evaluating the relationship between ovarian hormones and swallowing.

## Abbreviation Used

EMG: electromyography
TMJ: temporomandibular joint
